# RSCA genotyping of MHC for high-throughput evolutionary studies in the model organism three-spined stickleback *Gasterosteus aculeatus*

**DOI:** 10.1186/1471-2148-9-57

**Published:** 2009-03-16

**Authors:** Tobias L Lenz, Christophe Eizaguirre, Sven Becker, Thorsten BH Reusch

**Affiliations:** 1 Department of Evolutionary Ecology, Max Planck Institute for Evolutionary Biology, August-Thienemann-Str. 2, 24306 Plön, Germany; 2 Canada Centre for Inland Waters, Environment Canada, 867 Lakeshore Road, Burlington, Ontario, L7R 4A6, Canada; 3 Leibniz-Institute of Marine Sciences (IFM-GEOMAR), Evolutionary Ecology of Marine Fishes, Düsternbrooker Weg 20, 24105 Kiel, Germany

## Abstract

**Background:**

In all jawed vertebrates, highly polymorphic genes of the major histocompatibility complex (MHC) encode antigen presenting molecules that play a key role in the adaptive immune response. Their polymorphism is composed of multiple copies of recently duplicated genes, each possessing many alleles within populations, as well as high nucleotide divergence between alleles of the same species. Experimental evidence is accumulating that MHC polymorphism is a result of balancing selection by parasites and pathogens. In order to describe MHC diversity and analyse the underlying mechanisms that maintain it, a reliable genotyping technique is required that is suitable for such highly variable genes.

**Results:**

We present a genotyping protocol that uses Reference Strand-mediated Conformation Analysis (RSCA), optimised for recently duplicated MHC class IIB genes that are typical for many fish and bird species, including the three-spined stickleback, *Gasterosteus aculeatus*. In addition we use a comprehensive plasmid library of MHC class IIB alleles to determine the nucleotide sequence of alleles represented by RSCA allele peaks. Verification of the RSCA typing by cloning and sequencing demonstrates high congruency between both methods and provides new insight into the polymorphism of classical stickleback MHC genes. Analysis of the plasmid library additionally reveals the high resolution and reproducibility of the RSCA technique.

**Conclusion:**

This new RSCA genotyping protocol offers a fast, but sensitive and reliable way to determine the MHC allele repertoire of three-spined sticklebacks. It therefore provides a valuable tool to employ this highly polymorphic and adaptive marker in future high-throughput studies of host-parasite co-evolution and ecological speciation in this emerging model organism.

## Background

Natural genetic variation is the basic material for evolution, and to study its origin and persistence at the molecular level is required if we are to understand how species and populations evolved as a response to selection. The major histocompatibility complex (MHC) is one of the most polymorphic regions in the vertebrate genome [1,2]. Classical MHC genes (class I and II) encode for cell surface molecules that present self and non-self antigens to T-cells and therefore play an essential role for the recognition of pathogens invading the body [3]. MHC genes are also involved in mate choice decisions in several species [4]. The high polymorphism of MHC class I and II genes is reflected at three levels: (i) the presence of several gene loci, often as a result of recent duplication (ii) a high total number of alleles (iii) an exceptionally high nucleotide diversity at the sequence level between any two alleles. It probably results from natural selection due to co-evolving parasites, bacteria and viruses, and from sexual selection [5-8].

The population genetics of classical MHC genes has stimulated a growing initiative of research that is increasingly focussed on natural populations. Yet, its polymorphism also presents a serious challenge for the development of reliable genotyping methods. In many mammalian species it is nowadays possible to target single loci, because of a relatively stable structural organisation and gene orthology within the mammalian MHC due to ancient locus duplications [9,10]. In contrast, non-mammalian species show a substantially lower conservation in their MHC structure [9]. This leaves a large black box concerning the organisation of the MHC and gene orthology in most non-model species, and locus-specific typing is hardly possible (e.g. [11-15]). It is therefore important to our understanding of the MHC and its role in evolution and ecology that we find a reliable typing method for MHC diversity that can cope with large numbers of alleles but does not depend on detailed species-specific knowledge about the MHC organisation.

When using a PCR-based approach, the most reliable method for allele identification so far has been sequencing with prior allele separation via cloning of PCR products. However, this requires substantial effort, which increases exponentially with the expected number of alleles. As sequenced clones represent only a small sub-sample of the amplified fragments, PCR artefacts may lead to an overestimation of true allele number, in particular when only a small number of clones from each individual is sequenced to save resources [16,17].

**Figure 1:**
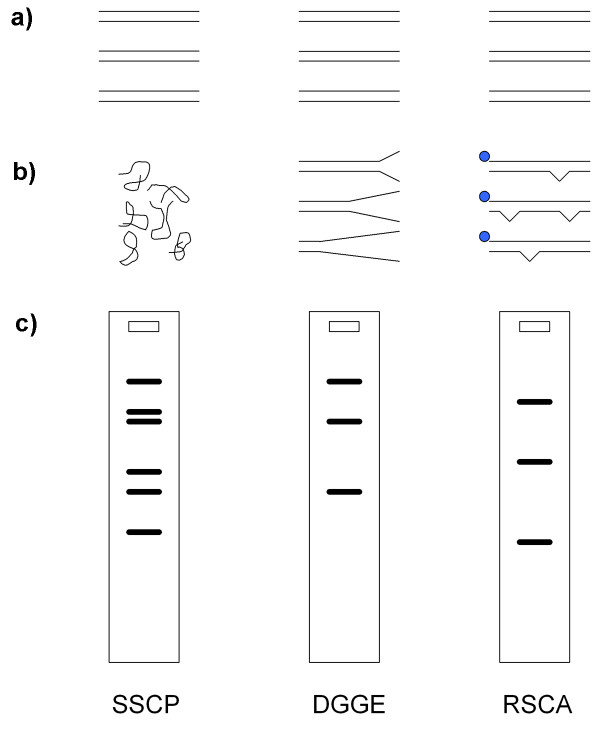
**Schematic differences between indirect genotyping techniques**. Commonly used indirect genotyping techniques: single-strand conformation polymorphism (SSCP), denaturing gradient gel electrophoresis (DGGE) and reference strand-mediated conformation analysis (RSCA). The starting material (a) is the same for all three techniques (PCR product with for instance three different sequence variants). SSCP: after denaturation, single strands form a sequence-specific structure (b). This structure leads to differential mobility in a non-denaturing matrix and two bands per variant (c). DGGE: the sequence-specific denaturation characteristics in a chemical gradient (in the gel) lead to partial separation of strands (b). This in turn leads to differential mobility and results in a single band per variant (c). RSCA: hybridisation with a labelled reference strand produces double-strands with sequence-specific mismatches (b). These mismatches lead to differential mobility in a non-denaturing matrix (c). Note that the gel with RSCA is shown for demonstrating reasons only. See method section for more details.

Faster and less expensive methods for genotyping of unknown alleles have been employed. The most common ones are denaturing gradient gel electrophoresis (DGGE) and single-strand conformation polymorphism analysis (SSCP) (both discussed in [18]). DGGE separates double-stranded sequence variants according to their denaturation characteristics and, under optimal conditions, provides a single band per variant (Fig 1). SSCP analysis achieves separation of variants due to mobility differences of the two complementary single strands in a non-denaturing matrix and can also be run in a capillary system (CE-SSCP [19]), yielding two bands, or peaks, per variant (Fig 1). Several factors can complicate allele identification with these methods. For instance, large numbers of distinct variants in the pool increase the likelihood of overlapping, indistinguishable peaks, or variation in the gel matrix between runs confounds the comparison of samples that have been run at different times.

A disadvantage of indirect typing methods is also the lack of sequence information, which is, however, imperative when studying MHC genes where only specific peptide binding residues are under positive selection [20,21]. While this lack of sequence information can be overcome by sequencing fragments separated on a gel matrix in low-throughput methods such as SSCP and DGGE, this is not possible for high throughput typing protocols such as CE-SSCP.

Reference Strand-mediated Conformation Analysis (RSCA [22]) is a PCR-based genotyping technique, which is increasing in popularity. Here, all sequence variants are amplified simultaneously and hybridised to a given reference strand. Upon cooling, heteroduplexes are formed with distinct mismatches for each variant. The reference strand is a known sequence variant, produced by amplifying a single allele template (plasmid or homozygous individual). These heteroduplexes are then separated according to their specific mobility in a non-denaturing environment, depending on the tertiary structure of each heteroduplex (Fig 1). This mechanism provides a significant advantage over other commonly used indirect typing techniques because it produces as many mobility values (bands or peaks) per allele as desired by using several distinct reference strands, creating a multi-dimensional coordinate for each allele. This enables differentiation of highly similar sequence variants [23].

Since its development by Argüello *et al. *[22], RSCA has been used in a number of species to type MHC loci, namely in humans (e.g. [24]), other primates (e.g. [25-28]) and several non-primate mammalian species (e.g. [29-33]). However, its application in non-mammalian species is still rare. To our knowledge the only studies so far have been performed in brown trout [34] and red jungle fowl [35], where only one and two loci have been addressed at a time respectively.

The three-spined stickleback *Gasterosteus aculeatus *is an emerging genomic model [36] that is increasingly used to study evolutionary phenomena, such as, sexual reproduction (e.g. [15,37,38]), host-parasite co-evolution (e.g. [39,40]), ecological speciation (e.g. [41-45]) and evolutionary developmental biology (e.g. [46,47]). In this study we focused on the class IIB genes of the stickleback MHC, which influence parasite resistance [39,48], mate choice [15,37,49], survival [50] and lifetime reproductive success [51].

Initially, six MHC class IIB loci in the stickleback have been reported [52], but this estimate was recently reduced to 2–4 [53]. Due to recent locus duplication and/or inter-locus recombination [54], it is not possible to target these loci individually. Therefore several alleles per individual have to be differentiated, independent of the genotyping technique used. This represents a strong challenge even for a strategy based on cloning, the gold standard genotyping method, due to the increased rate of sequence artefacts under certain conditions [17]. The established CE-SSCP protocol for the stickleback has been known to provide limited resolution due to a lack of sequence variant resolution [15]. We therefore developed a new genotyping protocol based on RSCA and a plasmid library of MHC class IIB alleles. We also determined the allele sequence represented by each RSCA allele peak. Results of the allele- and sequence-specific RSCA typing were verified by cloning and sequencing the MHC IIB genes of 23 individual fish.

## Methods

Here we describe in detail the different steps that are necessary to develop and apply an RSCA genotyping protocol to a new species. As an example we used the highly polymorphic MHC IIB genes of the three-spined stickleback *Gasterosteus aculeatus*. The exon 2 is the most variable region of these genes and encodes for the functionally important peptide binding grove of the MHC protein molecule [21]. For simplicity reasons we refer to different sequence variants as alleles, although they may originate from different loci.

### Primer design

We employed three criteria for RSCA primer design: (i) complementarity to a conserved region, including all possible sequences in the detected MHC allele pool, (ii) amplification of most of the highly variable exon 2 (iii) binding of both primers within the exon 2 to avoid any length variation of the amplicon. The presence of amplicons of different length may cause PCR conditions that outcompete longer amplicons [55], and in RSCA may lead to a hybridisation bias with the reference strand.

**Figure 2:**
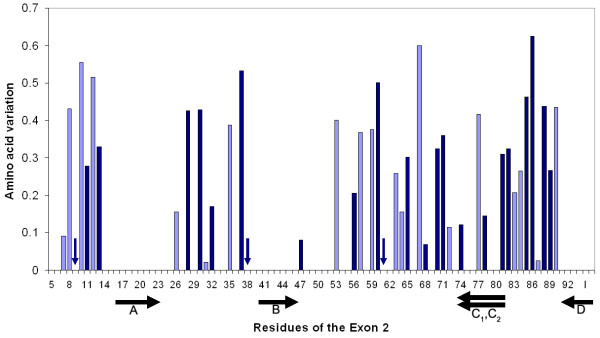
**Variation of amino acid residues in exon 2 of the MHC IIB genes**. Included are 31 sequence variants from local populations of three-spined sticklebacks (*Gasterosteus aculeatus*). The variation for each residue is based on the number and frequency of substitutions and is calculated as y = 1 - Valdar01 score, as determined with the Scorecons server by Valdar [64]. Dark bars and vertical arrows indicate residues involved in antigen binding, according to Brown *et al. *[21]. Black arrows below the panel show positions of the primers used for amplification of the Exon 2: A (GAIIEx2startF) and D (GAIIExon2R_RSCA) are the new primers designed for this study; B and C_1_, C_2 _are the primers used in previous CE-SSCP assessments of the stickleback MHC [15,19]. For visualisation, the variation plot was extended by 6 bases into the adjacent intron 2 (I, right side of panel). Despite the fact that an amino acid variation score cannot be calculated for an intron sequence, these 6 base pairs were fully conserved in all local sequences investigated so far.

Due to the high sequence variation in exon 2 of the MHC class IIB loci [52,53], the first criterion is a certain challenge with respect to the primer design. However, recently published sequence information [15,53,54] and the stickleback genome [56] provided good sources for the design of new primers. Additionally we designed a primer for the conserved exon 1 of the MHC IIB genes (GAIIEx1F: 5'-CAG CGT CTC CCT CCT CTT CAT-3') and cloned the exon 1 to intron 2 sequence of a number of fish to obtain sequence information about the so far rarely addressed beginning of the exon 2. Based on this, the new forward primer: GAIIEx2startF (5'-GTC TTT AAC TCC ACG GAG CTG AAG G-3') was then set in a fully conserved region at the beginning of exon 2 (Fig 2, arrow A) and is therefore optimised for the three-spined stickleback. The new reverse primer GAIIExon2R_RSCA (5'-ACT CAC CGG ACT TAG TCA G-3') spans the exon 2 – intron 2 boundary and lies in a conserved region as well (Fig 2, arrow D). The partial connection to the exon 2 avoids any length polymorphisms, which occur frequently in the rest of the intron 2. Although the new reverse primer bridges the exon-intron boundary, it can nevertheless be used for expression studies, because more than two thirds of it align within the exon 2 and the remaining one third is complementary to both the beginnings of intron 2 and exon 3, which are highly similar, leaving only 2 conserved mismatches at the 5'-end when used with reverse-transcribed cDNA. This new primer combination amplifies exon 2 in all currently known MHC IIB loci, produces a fragment of 247 base pairs (203 bp without primers) and spans 88% of the entire exon.

### Selection of RSCA reference alleles

**Figure 3:**
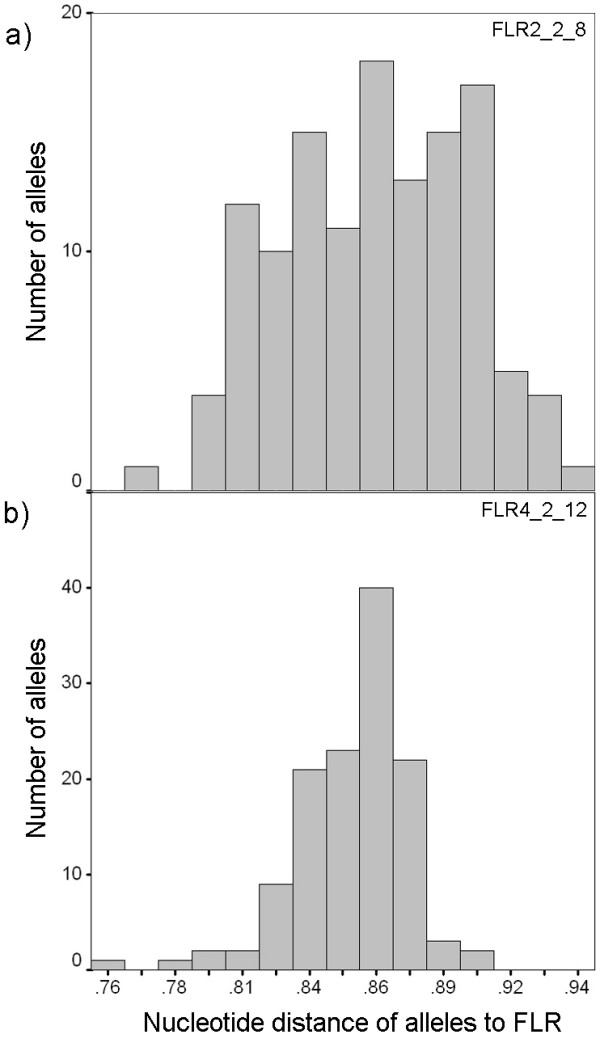
**Suitability of reference alleles (FLR)**. Distribution of genetic distances between alleles in the collected library and a) a suitable FLR with a broad distribution of genetic distances, b) a less suitable FLR with a narrow distribution. Distance is measured in pair-wise nucleotide p-distance. Note the different scale of the y-axis between panel a) and b).

The selection of suitable reference alleles is a crucial step in the optimization of RSCA. Selection criteria were: First, the reference alleles should represent sequence variants that do not occur in the screened populations. Second, they should not be too genetically distant to assure reliable hybridisation. And third, to increase resolution, the individual reference sequences should be as dissimilar from each other as possible. Due to the trans-species polymorphism of the MHC [57] it is even possible to use reference sequences from closely related species [25]. However, this increases the risk that certain alleles do not hybridize, which again increases with the complexity of the template (number of alleles) because of competition between alleles during the hybridisation reaction. Generally, when no sequence information is available, the resolution of the reference alleles can be tested by screening a subset of individuals, followed by a comparison of the peak distribution between references to choose the ones that show the broadest distribution and the highest number of peaks. For our study we tested nine cloned MHC IIB sequence variants of three-spined sticklebacks from a West Canadian population (TBH Reusch & T Reimchen, unpublished data), which due to long divergence time [58] are unlikely to carry any European alleles. Due to the existence of a database of more than 120 sequence variants that have been sequenced over the years [15,53,54] [TBH Reusch, KM Wegner, C Eizaguirre & TL Lenz, unpublished data], we were able to compare the genetic distance between the Canadian alleles and our local ones, to be able to choose candidate alleles that differed from each other more, but from the local alleles on average less than the population average. Additionally we estimated the distribution of the genetic distance between each reference and all the known alleles from our local populations to choose the flattest and broadest distribution (see Fig 3 for examples). A broad distribution promises the highest resolution of alleles, because genetic distance and molecule mobility in the gel matrix are highly correlated due to the three-dimensional structure of the heteroduplex, which increases in complexity with more mismatches between the reference and the target strand. A flat and broad distribution of the genetic distances between reference allele and the allele test pool therefore leads to a broader distribution of allele peaks and avoids overlap between them.

### General RSCA protocol

#### - Fluorescent labelled reference strands (FLR)

To enable high-throughput genotyping via capillary electrophoresis on an automated sequencer, the reference strands were fluorescently labelled. The templates for the FLRs were plasmids with a single sequence variant that were obtained by cloning three-spined sticklebacks from a Canadian population (TBH Reusch & T Reimchen, unpublished data). Three reference alleles were selected according to the criteria outlined above [GenBank:DQ016421, DQ016415 and FJ606785]. The plasmids were amplified with the same primer pair as the unknown alleles, except that the forward primer was labelled with fluorescein (FAM). The PCR mix for a 50 μl reaction volume contained 5 μl diluted plasmid (~10 ng/μl), 1x GeneAmp PCR Buffer II (Applied Biosystems), 5 mM MgCl_2_, 50 μM of each dNTP, 0.5 μM of each primer and 2.5 units of AmpliTaq Gold (Applied Biosystems). The following PCR program was used: 95°C for 10 minutes to activate the hot-start polymerase, 33 cycles of 94°C for 30 seconds, 58°C for 30 seconds and 72°C for 60 seconds with a final extension step of 72°C for 5 minutes. We ran our programs on the thermal cyclers PC-200 (Bio-Rad, Munich, Germany) or LabCycler (SensoQuest, Göttingen, Germany). The PCR products were purified with the NucleoSpin Extract II Kit (Macherey Nagel, Düren, Germany) and eluted in 100 μl HPLC grade water (Mallinckrodt Baker, USA). The purified FLRs were kept at -20°C until further use.

#### - Amplification and hybridisation

The newly developed primer pair GAIIEx2startF and GAIIExon2R_RSCA amplifies exon 2 of the MHC class II loci from genomic DNA extracted from ethanol-preserved muscle tissue of three-spined sticklebacks from our local populations. The exact origin and time of sampling is shown in Tab 1 [see Additional file 1]. For DNA extraction we used the DNeasy Blood and Tissue kit (Qiagen, Hilden, Germany). A PCR reaction volume of 25 μl contained ~90 ng of DNA, 1× GeneAmp PCR Buffer II, 5 mM MgCl_2_, 50 μM of each dNTP, 0.5 μM of each primer and 1 unit of AmpliTaq Gold polymerase. The following PCR program was used: 95°C for 10 minutes to activate the hot-start polymerase, 27 cycles of 94°C for 30 seconds, 58°C for 30 seconds and 72°C for 60 seconds with a final extension step of 72°C for 5 minutes. The low number of PCR cycles was chosen to avoid formation of PCR artefacts [17,59]. A reconditioning PCR step against artefact formation [60] was omitted in our protocol, because heteroduplexes that form after the last PCR cycle can be neglected due to the subsequent hybridisation reaction of target and FLR in the RSCA protocol.

Next, the appropriate amount of PCR product was mixed with the FLRs. This has to be adjusted for each FLR, because the hybridisation efficiency is altered by the average genetic distance and GC content between target alleles and FLR. The ratio depends also on the concentrations of the PCR product and the FLR. Eventually, a good ratio should give equal heights of homo- and heteroduplexes in RSCA. In our study we used 6 or 8 μl PCR product, depending on the FLR (1 μl each). The hybridisation started with denaturation at 95°C (10 min), then the heteroduplex formation is facilitated by a slow cooling of 2°/sec to 55°C, which is subsequently held for 20 minutes. A final cooling step at 4°C (15 min) assures stabilisation of the heteroduplexes. This hybridisation product is stable for several hours at 4°C and for several days at -20°C.

#### - Capillary electrophoresis

The separation of heteroduplexes was performed on a model ABI PRISM 3130xl Genetic Analyzer (Applied Biosystems) with a 36 cm capillary. The Conformation Analysis Polymer (CAP, Applied Biosystems) provided the non-denaturing matrix in which the heteroduplexes migrate according to their tertiary structure. We used a 5% polymer: 5 g 9% CAP (used at room temperature), 2.16 g Urea (Sigma-Aldrich, Steinheim, Germany), 0.95 g HPLC grade water and 0.9 g 10× running buffer (Applied Biosystems). For each sample 1.5 μl of the hybridisation product was mixed with 0.3 μl GS1000 Rox size standard (Applied Biosystems) and 9.7 μl HPLC grade water. The internal size standard ensures proper alignment of heteroduplex peaks and minimizes between-run variation. The running conditions were: 18°C run temperature, 15 kV injection voltage, 15 sec injection time, 10 kV run voltage. The hybridisation products of each FLR had to be run separately, otherwise the antisense strand of one FLR would hybridise with the labelled strand of another FLR and produce a heteroduplex peak that cannot be differentiated from real alleles.

### Plasmid library for sequence-specific genotyping

A plasmid library of MHC class II exon 2 sequence variants has been collected over the years from cloned three-spined sticklebacks (TBH Reusch, KM Wegner, C Eizaguirre & TL Lenz, unpublished data). It comprises currently 83 distinct variants that differ to varying extents (1 to 47 of 203 bp). Of the 3,403 possible allele combinations in a pairwise comparison, only 0.7% differ by less than 3 bp (17 by 1 bp and 8 by 2 bp). We recorded mobility values for each sequence variant with each of the three chosen FLRs. This was done four times independently, spread over several months. To simulate realistic laboratory conditions, we used different polymer lots, the most likely source of between-run variation, and a capillary that is in turns used also for fragment analysis with POP-4™ polymer (Applied Biosystems). To estimate the resolution and specificity of the new typing method, we compared the difference in mobility values of alleles pair-wise in all 3,403 possible combinations. Those allele pairs that differed for all three chosen FLRs in their mean mobility values by less than their combined standard deviation were assigned to be undistinguishable.

### RSCA typing procedure

MHC IIB exons 2 of individual three-spined sticklebacks were amplified with the newly developed primers and processed according to the protocol outlined above. Mobility values for each heteroduplex peak were recorded after alignment of the internal size standard. Using the mobility values from the three FLRs and the library with mobility values of the collected alleles, we assigned allele identity to the heteroduplexes [see Additional file 1: Fig 5 for an example]. However, the identity of an allele was only assigned if all three corresponding mobility values (+/- 1 SD) from the library were found in the individual. In cases where not all three values from the allele library could be found in an individual, we marked the heteroduplex peak as new allele. Individuals with such unknown heteroduplexes were subsequently cloned to identify the sequence of the new allele.

### Cloning of MHC genes in selected fish to verify RSCA typing

To verify our RSCA genotyping protocol, we compared it with the result of 23 sticklebacks from different locations and time points, whose MHC IIB genes were cloned and sequenced. The cloning for ten of these fish (all from a single lake population) had been done in a former study to establish a reliable amplification and cloning protocol for multi-locus templates [17]. That study involved steps to avoid artefact formation during PCR and the sequencing of on average 89 clones per individual. That study still used a former, at that time established primer pair for exon 2. However, it is important to note that in this lake population the former primer pair and the newly designed primer pair from this study amplify the same alleles. It is therefore safe to employ these comprehensive cloning results for validation of the RSCA protocol. We also cloned the MHCIIB genes of an additional set of 13 three-spined sticklebacks from different populations, some of which, according to RSCA typing possessed alleles that were not present in the plasmid library. For the amplification and cloning of their MHC genes, we followed the protocol in Lenz & Becker [17], but with the new primer pair developed for this study (see above), two independent amplification reactions and 48 clones per fish for sequencing. Forty-eight clones is a threshold that we determined by applying accumulation curves on data from the first cloning set [17], which resulted in 99.99% probability to have typed all present sequence variants (data not shown).

### Data analyses

For alignment and estimation of genetic distance between sequences, we used BioEdit 7 [61]. RSCA chromatograms were aligned along the peaks of the internal size standard and analysed with GeneMarker 1.6 (SoftGenetics, PA State College, USA). Due to the non-denaturing feature of the polymer, the manufacturer's established values of the size standard did not match the peak pattern in our analysis, therefore the values for the size standard peaks were assigned new, starting with 1,000 arbitrary units for the longest fragment. The software Primer 6 [62] was used to calculate accumulation curves based on clone data from a previous cloning project [17]. To obtain a bootstrap estimate of confidence, we computed 999 times the clones from two individuals with five alleles. MEGA 4 [63] provided the d_N_/d_S _ratio and z-test for positive selection. We used the ScoreCons online server [64] to determine variation for amino acid residues of the exon 2. The software MultiLocus 1.22 [65] was used to estimate linkage disequilibrium (I_A _– association index) between detected alleles, and 1,000 randomizations were run.

## Results

### RSCA typing of plasmid library

**Figure 4:**
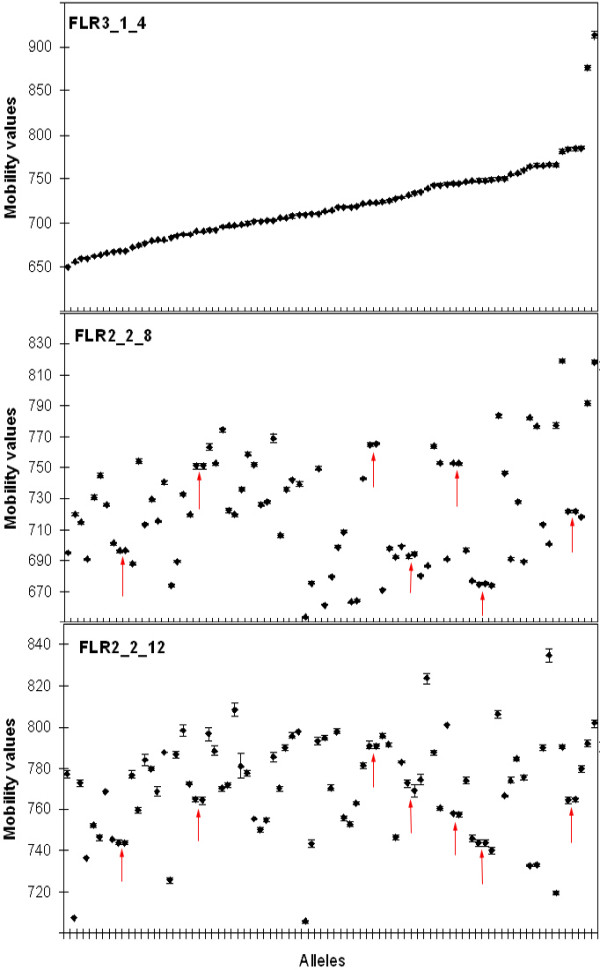
**Mobility values of 83 alleles with the selected three reference alleles (FLR)**. Mean and SD of each allele from four independent runs are shown. Arrows indicate the allele pairs that can not be differentiated with the three FLRs. Alleles are presented in the order of mobility with the reference FLR3_1_4 (top panel).

The three mean mobility values for each of the 83 allele variants averaged over four independent runs are shown in Fig 4. To estimate the resolution of the RSCA typing method, we compared all 83 alleles pair-wise. The mean mobility difference between alleles was 46.5, 42.3 and 27.3 units for the three FLRs. In comparison, the average standard deviation of the independently obtained mobility values of an allele was 1.2, 1.0 and 1.4 units. In seven (0.2%) of 3,403 possible allele pairs, the two alleles were not distinguishable according to our definition outlined above. Six of them differed by 1 bp and one by 2 bp. The other 11 pairs with 1 bp and seven with 2 bp difference were distinguished unambiguously by using the three different FLRs (Fig 4).

### RSCA typing of MHC IIB genes in selected fish

By RSCA typing the MHC IIB genes of 23 fish, we detected 28 distinct sequence variants, 15 of which occurred at least twice in different individuals. The number of alleles per fish ranged from 2 to 5 with a median of 4 [Additional file 1: Tab 1]. Using the allele library, the mobility values of 23 alleles were identified unambiguously, and therefore their sequences were determined [Additional file 1: Tab 1]. Five alleles were assigned as new according to our stringent selection criteria (see Methods section), and because their mobility value combination was not identified with the existing allele library. Four individuals carrying these five new alleles were therefore subjected to cloning to identify the sequence of the new alleles.

### Comparison between cloning and RSCA typing

Cloning and sequencing of the same 23 individuals that were used for RSCA typing revealed 27 distinct alleles. All sequences showed high similarity to stickleback MHC IIB exon 2 variants in a NCBI-BLAST search. Sixteen of the 27 alleles have been deposited already in GenBank [Additional file 1: Tab 1], and the remaining ones have been submitted during this study [GenBank:FJ360531 – FJ360541]. An additional very divergent sequence variant [GenBank:AF395709], which has been described before [53], was detected by cloning and RSCA typing in every individual investigated. Due to sequence conservation, this variant was not addressed in this study. This sequence, which can also be found in the genome sequence of the Alaskan stickleback, potentially originates from an invariant MHC locus that may have antigen processing function, similar to the invariant H2-M locus in mice [66].

The remaining alleles differed in 1 to 46 nucleotides with a mean of 26, and in 1 to 27 amino acids (mean = 15.7; d_N_/d_S _ratio = 1.96; Z-test, p = 0.018). The results of the cloning confirmed the previous RSCA typing in 22 out of the 23 individuals for allele number and allele identity, i.e. known alleles were recognised and unknown alleles were assigned as new and differentiated from each other. In one individual, a known allele (Neu51) was not recognised and assigned as new, although it was present in the allele library and had even been recognised in another fish of the same set [Additional file 1: Tab 1]. This finding was resolved by following the proposed protocol, i.e. cloning the respective individual to resolve the possible new sequence variant. In this set of 23 individuals therefore 16,240 bases were typed correctly and another 1,218 bases (5 different alleles in 4 individuals) were scheduled for cloning by the proposed protocol.

The detected alleles were in strong linkage disequilibrium (I_A _= 0.38, p < 0.001). All alleles that occurred in more than one individual belonged to specific haplotypes with one to three variants per linkage group [Additional file 1: Tab 1]. In total, 15 distinct haplotypes were differentiated, two of which shared one allele.

We also re-typed the first ten fish, which had been cloned with a different primer pair in the earlier project [17], with RSCA using the same former primer pair to enable direct comparison between methods. The same number of alleles per individual was observed with both methods, which shows that the RSCA typing protocol is reliable, independent of the primer pair used.

## Discussion

In this study we developed a protocol for reliable genotyping of polymorphic multi-copy genes, using the highly polymorphic MHC class IIB genes of three-spined sticklebacks (*Gasterosteus aculeatus*) as an example. The new high-throughput genotyping protocol is based on Reference Strand-mediated Conformation Analysis (RSCA). To verify the results obtained from RSCA typing, we cloned and sequenced a total of 23 individuals to saturation. The congruence between RSCA and cloning in number as well as identity of detected alleles shows the reliability of the new typing protocol.

While cloning and sequencing is time consuming and laborious, our RSCA protocol can handle a high number of fish in shorter time. Incorporating an extensive library of sequence variants from wild populations, we were able to unambiguously identify the sequence information corresponding to each fluorescent signal, or alternatively, to assign new alleles if applicable. Only in one case was an allele erroneously assigned as new, although its sequence was already known. This is a result of the adopted stringent typing procedure and represents a conservative error, which was resolved by following the typing protocol, i.e. cloning and sequencing of MHC in this individual.

A prerequisite to determine the sequence represented by each allele peak is a comprehensive plasmid library. To employ the proposed RSCA protocol for a new species with the intention to not only distinguish between distinct alleles but to identify alleles at the sequence level, such a library has to be established by cloning and sequencing of unknown alleles retrieved from the population. This strategy is less laborious than to subject all individuals to cloning and sequencing.

The relatively high number of individuals with unknown alleles presented here (22% carried one or more new alleles) is due to the fact that several of these individuals were chosen for cloning, because they contained new alleles. In pilot screens of local populations (C. Eizaguirre & T.L. Lenz, unpublished data), the average fraction of individuals with new alleles ranged from 5–7%, i.e. only this fraction of a given sample set has to be cloned to obtain complete sequence information for all individuals. Evidently, the more complete the underlying allele library, the lower the fraction of fish with novel alleles.

It remains to be tested whether primers developed based on North European fish will work satisfactorily across all stickleback populations from the Northern hemisphere, considering the divergence between European populations [58] and even more so between the Atlantic and the Pacific clades [67]. The currently available MHC sequence information from the genome of an Alaskan individual and sequences of some individuals from British Columbia/Canada (TBH Reusch & T Reimchen, unpublished data), however, support the universality of our new primers. Using these primers in the current study, we found a median of 4 alleles over 23 individuals, which is slightly lower than the previously reported 5.8 alleles per individual detected by CE-SSCP [68]. This might be due to the limited number of individuals in this study, but it might also indicate that the previously used combination of two reverse primers [15] slightly overestimated the total MHC diversity. This would be in agreement with a recent finding, which estimates the number of MHC class IIB loci in the three-spined stickleback to be only 2–4 [53].

Since gene expression determines the phenotype, it is an important aspect to know how loci are expressed, which also affects the interpretation of allele and sequence data. The extraction of RNA and the reverse-transcription to cDNA are more laborious than the extraction of genomic DNA, but getting an estimate on locus expression at least in a subsample of individuals would be desirable when investigating new species. Here, we did not test whether any detected alleles are expressed at the mRNA level. However, a previous screen of several stickleback families revealed that over 90% of the alleles detected by CE-SSCP are expressed [39], and there is no reason why this should not apply to the alleles detected by RSCA. In combination with the new typing technique, it remains to be tested whether all alleles are expressed in all organs of an individual, and whether there is locus-specific transcription regulation.

The first of the two only studies employing RSCA in non-mammalian species so far directly compared RSCA with SSCP typing, when genotyping the MHC class IIB locus in lake trout (*Salvelinus namaycush *[34]). In the end, the authors favoured SSCP over RSCA, because it detected some additional alleles in the screened population. However, the existence of these additional alleles was not verified via sequencing. Moreover, the lake trout carries only one MHC IIB locus, which limits the number of detectable alleles to two per individual. The authors of the second study developed an RSCA typing protocol for both MHC class I and II in the red jungle fowl (*Gallus gallus*) and addressed two loci at a time respectively [35]. In concordance with our study, it was concluded that RSCA is a reliable technique for MHC typing in the red jungle fowl.

In many species, the MHC consists of several loci in classes I and II [69], which makes the allele pattern more complicated and increases the chance of overlapping signals using SSCP, a problem that occurred already with the single locus in the Lake trout [34]. Here, RSCA has a substantial advantage over other indirect typing techniques, because it provides several mobility values per allele, reducing the chance of two overlapping allele values and thus leads to increased resolution.

Reproducibility is also a major concern for indirect genotyping techniques and can be confounded by polymer lot variation, temperature fluctuation and other factors [70]. Therefore we measured the variation of mobility values for each allele between independent runs over several months. By this we were able to show that between-run variation with RSCA is limited and can again be overcome by obtaining several mobility values from the different labelled references. We conclude that three different well-chosen reference strands are sufficient to differentiate more than 99% of all allele pairs. To employ more reference alleles would probably also differentiate the last alleles pairs, but we consider a resolution of 99% as satisfactory. Nevertheless, regular tests of known alleles/allele libraries are advisable to keep the mobility values "up to date" and counteract unannounced chemistry changes by suppliers.

A new observation due to the high resolution in allele detection by RSCA is the fact that alleles seem to occur in linked haplotypes, differ in the number of sequence variants per haplotype and in one case share an allele between haplotypes. This provides a hint for strong linkage disequilibrium between loci, and low but yet occurring recombination in nature, an observation that is in agreement with previous results on the structure of the MHC region in the stickleback [54]. A recent model for Associative Balancing Complex evolution (ABC model [71]) proposed that strong linkage disequilibrium around MHC loci could result in balancing selection by hitchhiking deleterious mutations and might contribute to the maintenance of MHC polymorphism. This is an interesting idea, albeit one of the prerequisites for this model is polymorphism itself, both in terms of number of alleles and gene diversity under which the recessive mutations are maintained as a 'sheltered load' [71]. The polymorphism in the MHC must therefore – at least initially – have originated from a different selective pressure, such as host-parasite co-evolution [7], but ABC evolution could potentially contribute to its maintenance.

The detected difference in the number of alleles between haplotypes might indicate variation in the number of loci between haplotypes and addresses a phenomenon that has already been described for other species [72-74]. This finding would explain the earlier reported large variance in allele numbers among individuals [68], and it reveals a potential mechanism of adaptation to changing pathogenic environments, which has first been termed by Klein *et al. *[75] as '*The Accordion Model of MHC Evolution*', and later was elaborated by Nei *et al. *[10] to the Birth-and-Death-Model of Evolution. Nevertheless, these findings need further research at the genomic level, including more thorough analysis of the haplotype-specific chromosomal organisation.

## Conclusion

Here we present a new RSCA genotyping protocol for the highly polymorphic MHC genes of the three-spined stickleback *Gasterosteus aculeatus*, which in combination with an established allele library provides sensitive and reliable allele data at the sequence level. Verification of the RSCA typing by cloning and sequencing shows high congruency between both techniques. Together with new insights from the polymorphic MHC of the three-spined stickleback, an emerging model system, this offers a resource for researchers to address questions of host-parasite co-evolution, local adaptation and ecological speciation.

## Authors' contributions

TLL, CE, SB and TBHR designed the study. TLL carried out the molecular work. CE participated in the cloning and sequence analysis. TLL and CE performed the statistical analysis. TLL drafted the manuscript. CE, SB and TBHR helped to draft the manuscript. All authors read and approved the final manuscript.

## Supplementary Material

Additional file 1 **RSCA typing of MHC in stickleback.** – Table with samples and genotyping data. – Example figure of genotyping procedure. Click here for file
