# Supplementary material for: RSCA genotyping of MHC for high-throughput evolutionary studies in the model organism three-spined stickleback Gasterosteus aculeatus
Source: BMC Evol Biol. 2009 Mar 16;9:57. doi: 10.1186/1471-2148-9-57 (PMC2662802; doi:10.1186/1471-2148-9-57)
Supplement: Additional file 1 — RSCA typing of MHC in stickleback. – Table with samples and genotyping data. – Example figure of genotyping procedure. [file 1471-2148-9-57-S1.pdf]

Additional file 1 – Lenz et al., RSCA typing of MHC in stickleback

Table 1: Three-spined stickleback individuals typed for comparison of RSCA and cloning. Given are Fish ID, the population and year in which they were caught, alleles as determined by RSCA and cloning, match between both typing methods and known accession numbers in GenBank. Note the strong linkage with one to three alleles per haplotype.

Fish ID	Population/Year	RSCA	Cloning	Match	AccessionNo
MM04031	GPS/2004	No01	No01	✓	DQ016399
		No12	No12	✓	DQ016400
		No15	No15	✓	DQ016410
		No16	No16	✓	DQ016417
MM04039	GPS/2004	No13	No13	✓	AF395711
		No18	No18	✓	AY687846
MM04047	GPS/2004	No01	No01	✓	DQ016399
		No12	No12	✓	DQ016400
		No13	No13	✓	AF395711
		No18	No18	✓	AY687846
MM04050	GPS/2004	No13	No13	✓	AF395711
		No18	No18	✓	AY687846
		So05	So05	✓	DQ016402
		So11	So11	✓	DQ016404
		SCX03	SCX03	✓	AJ230191
MM04060	GPS/2004	No01	No01	✓	DQ016399
		No12	No12	✓	DQ016400
		No13	No13	✓	AF395711
		No18	No18	✓	AY687846
MM04080	GPS/2004	No13	No13	✓	AF395711
		No18	No18	✓	AY687846
		No08	No08	✓	AY687842
		SCX15	SCX15	✓	EU541449
MM04083	GPS/2004	No15	No15	✓	DQ016410
		No16	No16	✓	DQ016417
MM04086	GPS/2004	No01	No01	✓	DQ016399
		No12	No12	✓	DQ016400
		No10	No10	✓	AF395722
		No11	No11	✓	AY687843
MM04087	GPS/2004	No01	No01	✓	DQ016399
		No12	No12	✓	DQ016400
		So05	So05	✓	DQ016402
		So11	So11	✓	DQ016404
		SCX03	SCX03	✓	AJ230191
MM04131	GPS/2004	No01	No01	✓	DQ016399
		No12	No12	✓	DQ016400
		No08	No08	✓	AY687842
		SCX15	SCX15	✓	EU541449
R027	MA/2005	So06	So06	✓	FJ360531*
		So05	So05	✓	DQ016402
		SCX03	SCX03	✓	AJ230191
		So11	So11	✓	DQ016404
R110	MA/2005	No05	No05	✓	AY687829
		Neu51	Neu51	✓	AY687833
		SCX20	SCX20	✓	FJ360541*

Fish ID	Population/Year	RSCA	Cloning	Match	AccessionNo
S2MA07005	MA/2007	So02	So02	✓	DQ016426
		So05	So05	✓	DQ016402
		SCX03	SCX03	✓	AJ230191
		So11	So11	✓	DQ016404
S2MA07061	MA/2007	So06	So06	✓	FJ360531*
		So01	So01	✓	FJ360535*
		So10	So10	✓	FJ360534*
S2MA07066	MA/2007	No05	No05	✓	AY687829
		No42	No42	✓	FJ360536*
		new5	No45	new	FJ360537*
En07284	GPS/2007	No08	No08	✓	AY687842
		SCX15	SCX15	✓	EU541449
		No13	No13	✓	AF395711
		No18	No18	✓	AY687846
TL06121	GPS/2006	No05	No05	✓	AY687829
		No01	No01	✓	DQ016399
		No12	No12	✓	DQ016400
TL06165	GPS/2006	No08	No08	✓	AY687842
		SCX15	SCX15	✓	EU541449
		No15	No15	✓	DQ016410
		No16	No16	✓	DQ016417
E10 [1]	GPS/2005	No08	No08	✓	AY687842
		SCX15	SCX15	✓	EU541449
		new4	Neu51	⊗	AY687833
		SCX20	SCX20	✓	FJ360541*
E47	GPS/2005	No01	No01	✓	DQ016399
		No12	No12	✓	DQ016400
		No13	No13	✓	AF395711
		new1	No46	new	FJ360538*
E54	GPS/2005	No01	No01	✓	DQ016399
		No12	No12	✓	DQ016400
		No13	No13	✓	AF395711
		new1	No46	new	FJ360538*
E62	GPS/2005	No01	No01	✓	DQ016399
		No12	No12	✓	DQ016400
		No43	No43	✓	FJ360532*
		No44	No44	✓	FJ360533*
E77	GPS/2005	No15	No15	✓	DQ016410
		No16	No16	✓	DQ016417
		new2	No48	new	FJ360539*
		new3	No49	new	FJ360540*
No of distinct alleles		28 [1]	27		
No of alleles typed		86	86		

[1] In one fish (E10), the allele Neu51 was determined as 'new' by RSCA typing, although it was present in the plasmid library. The same allele was correctly identified in another fish (R110).
* Alleles submitted during this study. GPS – Großer Plöner See, MA – Malenter Au.

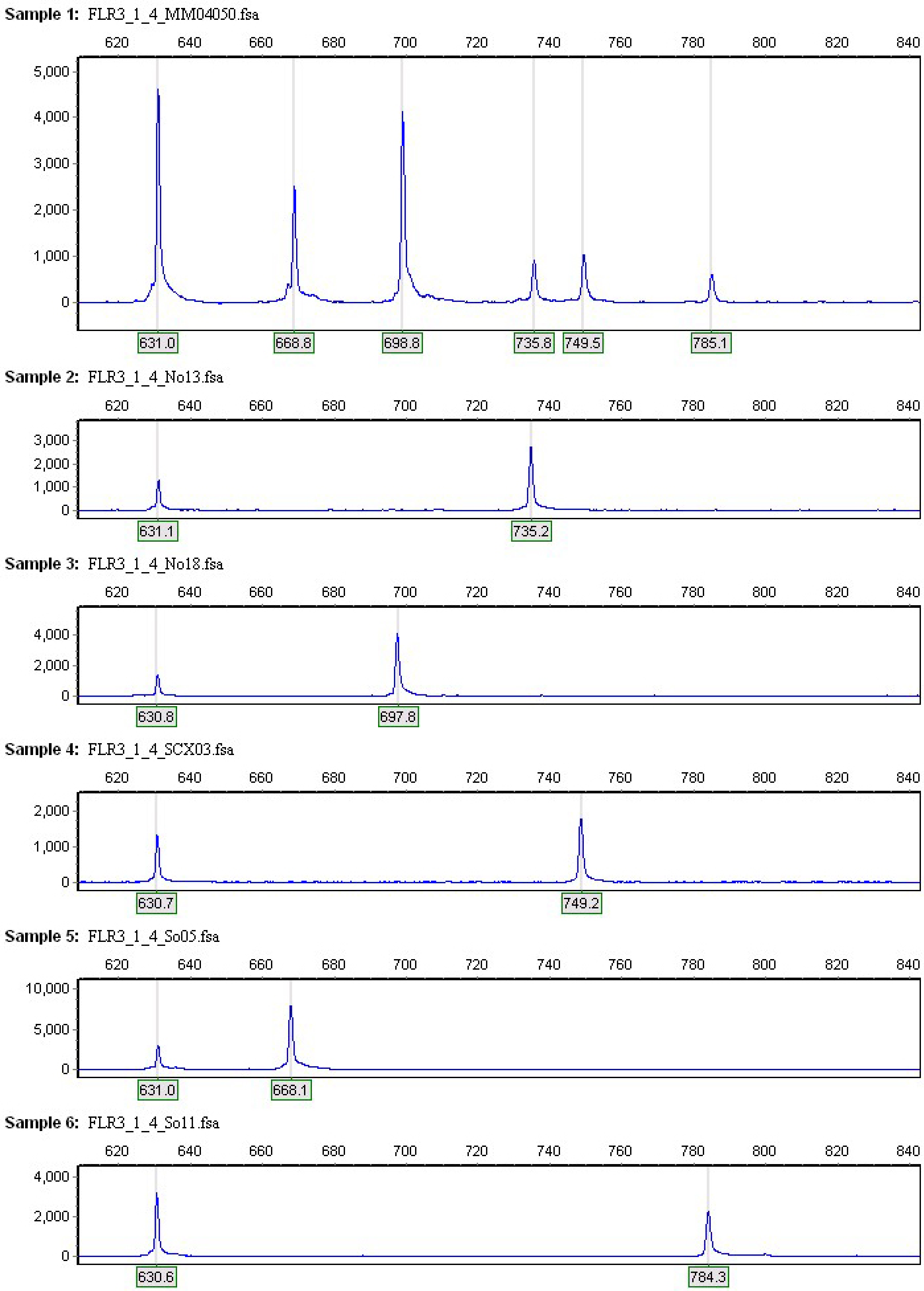

Figure 5: RSCA chromatograms. Top panel: typing of an individual with 5 alleles hybridised to one (of three) FLR. Panels below: five plasmids from the allele library hybridised to the same FLR. Each chromatogram shows the homoduplex of the FLR (leftmost peak) and five (top panel) or one (panels below) heteroduplex(es) for the individual alleles.
